# CHO-DHFR cell line development platform: Application of Clonepix and Automated Mini Bioreactor (AMBR) technologies to meet accelerated timelines

**DOI:** 10.1186/1753-6561-9-S9-P52

**Published:** 2015-12-14

**Authors:** Venkata R Mangalampalli, Dyane Wycuff, Mingzhong Chen, David Berlinger, Elizabeth H Scheideman, Amritha Menon, Guilia Fabozzi, Althaf Hussain, Richard M Schwartz

**Affiliations:** 1 Vaccine Production Program, Vaccine Research Center, NIAID, National Institutes of Health, 9 W Watkins Mill Road, Gaithersburg, MD - 20875, USA

## Background

The Holy Grail sought by all Bioprocess Cell Line Development (CLD) groups is achieving high yields from easily-cultured, robustly-growing cells in timelines measured in weeks rather than months. As the first bottleneck in process development, CLD must first birth its product for upstream and downstream groups to initiate their own reproductive cycles. To facilitate shortened CLD timelines, scientists have turned to new technologies and automation platforms. Emerging high-throughput instrumentation such as Clonepix and Automated MicroBioreactors (AMBR) have been enthusiastically integrated into stable cell line generation platforms; however, application of these methodologies among users is divergent.

In this study, we describe method development that is part of an ongoing effort to create a vigorous, adaptable, high-throughput cell line development platform, incorporating in-house VRC-DG44-CHO cells, expression vectors with high-activity regulatory elements, and efficient selection and evaluation methods that result in early generation of optimal-yielding, vigorous, stable cell lines. This study was aimed toward a) evaluating early incorporation of Clonepix technology to maximize transfectant pool heterogeneity, b) identifying Clonepix fluorescence attributes that increase the probability of high-productivity clone isolation, c) evaluating and optimizing AMBR operating conditions to achieve process attributes comparable to and predictive of medium-scale bioreactors, and implementation of these practices for maximal results with shortened timelines.

## Materials and methods

Host Cell Line: Adherent, serum-dependent CHO-DG44 cells were adapted to grow in suspension in serum-free, chemically defined production medium (ActiCHO P). Suspension-adapted cells were cloned using semi-solid clone CHO clone media and ClonePix II™ methodology. Isolated clonal cultures were assessed for growth traits, and the top 20 clones with fast growing profiles were pooled to comprise the VRC CHO DG44 host cell line.

Expression plasmid: One VPPL-DHFR vector, presenting two open reading frames for concomitant expression of heavy and light chains under distinct CMV promoters with DHFR expression in cis for selection for CHO DG44 cells, was assembled. Two heterologous enhancer elements have been inserted upstream of each CMV promoter to augment protein expression levels.

Cells were transfected with pre and post-optimized versions of VPPL-DHFR vector expressing mAb. Stable pools generated from traditional large pool (LP) selections were seeded at 1000 cells/mL in semi-solid media, while direct cloning (DC) cells at 48 hours post-transfection were seeded at 5000 cells/mL.

## Results

To streamline and maintain medium composition homogeneity from cell line development all the way up to production scale, we adapted our host cell CHO DG44 line to Acti-CHO P production medium by gradually weaning the cells from serum-containing medium to the ActiCHO SM medium, a leaner sibling of ActiCHO P production medium. When cells were completely serum free (theoretically calculated) and further passaged in ActiCHO SM medium, the cell doubling time was >57 h. To select a rapidly-cycling population, cells growing in Acti-CHO SM medium were plated in Clonepix semisolid medium, and individual clones were picked and seeded in 96 well plates and expanded to shake flasks. Top 20 clones with doubling times of 24 + 6 h were pooled in two pools, each of which demonstrated transfection efficiences of > 50% and doubling times of 24 + 4 h.

To reduce the cell line generation timeline, we evaluated Clonepix II technology implemention within 48 h post transfection compared to 3 weeks for pool generation followed by seeding semi-solid medium. Data supported that direct cloning in semisolid medium with 100 nM MTX at 48 hours post-transfection could reduce the CLD timeline to <3 months. Observable morphologies of the clones seeded 48 h post transfection were comparable to those of cells seeded from traditional work flow (1.0). We have also evaluated whether there is any correlation between Clonepix attributes and eventual high-producing clones.

**Figure 1:**
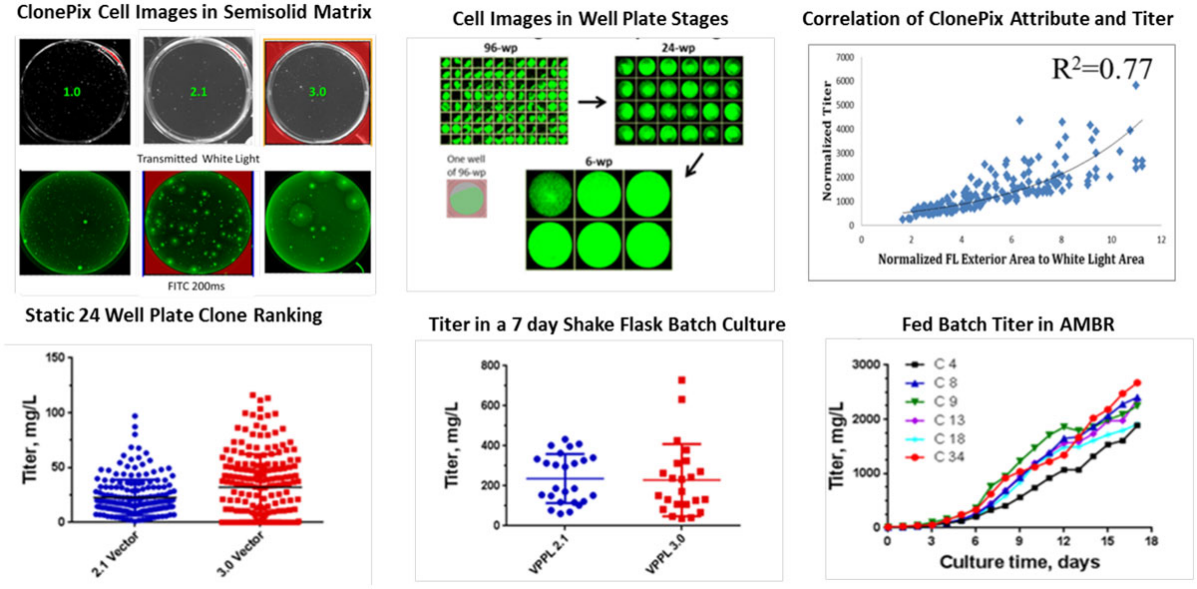
**Data from various stages of the CLD process flow**.

Our data indicated that Clonepix attribute such as normalized external fluorescence area to white light area were predictive of productivity at 96-well stage and beyond. Further clones were expanded from 96-well plates to shake flasks by down-selecting at each stage based either on confluence (select clones > 30% confluence) or titer. Our initial experiments with AMBR bioreactors for down-selecting the clones examined scalability to production scale bioreactors, used baseline operating conditions of aeration 0.11 slpm, agitation 700 rpm (up draft) with a calculated tip speed of 0.417 m/sec and gas velocity of 0.14 m/sec and maintains a KLa of 3 sec-1 for a 15 mL working volume, and resulted in inability to maintain DO at set 50% after day 5, indicating that the selected clones have high oxygen demands. Experiments to optimize operating conditions by changing the aeration and agitation rates to meet the high OUR (OTR) revealed that aeration of 0.27 slpm, agitation rate of 1000 rpm (down draft) with a calculated tip speed of 0.596 m/sec and gas velocity of 0.34 m/sec maintained a KLa of 7 sec-1, supplying the oxygen demand of these culture and maintaining DO at the 50% setpoint. Our top clones in optimized AMBR bioreactors produced about 2.6 g/L in a 17-day fed batch culture (Figure 1).

## Conclusions

Our data suggested that Clonepix attributes such as External fluorescence Area (EFA), White Light Area (WLA), and normalized EFA to WLA were predictive of productivity at the 96-well stage and beyond. Early AMBR optimization studies revealed that down-draft agitation maintained the dissolved oxygen (DO) concentration at higher cell densities than updraft agitation, maintained DO in the range of 50+5%, and resulted in clones with improved titers that were comparable to and predictive of those from 3 L bioreactors. Overall, our first generation integrated platform, comprising preadaptation of the host cell line to production medium prior to transfection, ClonePix technology introduction early in the process flow (48 hours post-transfection), and clone screening in AMBR for down-selection to the top three to five clones for 3 L bioreactor evaluation enabled us to generate stable clones at the AMBR stage with productivities of 2.5 to 3 g/L in a 14-day, fed-batch process. This integrated strategy reduced the CLD timeline to three months from vector generation to final clone selection.

